# GOGOT: a method for the identification of differentially expressed fragments from cDNA-AFLP data

**DOI:** 10.1186/1748-7188-2-5

**Published:** 2007-05-30

**Authors:** Koji Kadota, Ryoko Araki, Yuji Nakai, Masumi Abe

**Affiliations:** 1 Graduate School of Agricultural and Life Sciences, The University of Tokyo, 1-1-1 Yayoi, Bunkyo-ku, Tokyo 113-8657, Japan; 2 Transcriptome Research Center, National Institute of Radiological Sciences (NIRS), 9-1, Anagawa-4-chome, Chiba-shi 263-8555, Japan

## Abstract

**Background:**

One-dimensional (1-D) electrophoretic data obtained using the cDNA-AFLP method have attracted great interest for the identification of differentially expressed transcript-derived fragments (TDFs). However, high-throughput analysis of the cDNA-AFLP data is currently limited by the need for labor-intensive visual evaluation of multiple electropherograms. We would like to have high-throughput ways of identifying such TDFs.

**Results:**

We describe a method, GOGOT, which automatically detects the differentially expressed TDFs in a set of time-course electropherograms. Analysis by GOGOT is conducted as follows: correction of fragment lengths of TDFs, alignment of identical TDFs across different electropherograms, normalization of peak heights, and identification of differentially expressed TDFs using a special statistic. The output of the analysis is a highly reduced list of differentially expressed TDFs. Visual evaluation confirmed that the peak alignment was performed perfectly for the TDFs by virtue of the correction of peak fragment lengths before alignment in step 1. The validity of the automated ranking of TDFs by the special statistic was confirmed by the visual evaluation of a third party.

**Conclusion:**

GOGOT is useful for the automated detection of differentially expressed TDFs from cDNA-AFLP temporal electrophoretic data. The current algorithm may be applied to other electrophoretic data and temporal microarray data.

## Background

Expression analysis based on comparison of one-dimensional (1-D) electrophoretic patterns is one of the few genome-wide approaches that don't require sequence information. There are a few methods such as differential display [1], amplified fragment length polymorphism (AFLP) [2], and its variants like cDNA-AFLP, an AFLP-derived technique for RNA fingerprinting [3]. The cDNA-AFLP method and related techniques such as HiCEP have been widely used for gene discovery and monitoring temporal expression changes of transcript-derived fragments (TDFs) by comparing sets of time-course electropherograms [4-15]. However, inaccurate DNA fragment sizing often interferes with high-throughput analysis.

A major source of incorrect estimation of fragment lengths is the use of wrong size marker peaks when the true peaks are masked by intense peaks nearby [12,16]. Such electropherograms are locally expanded or compressed and the deviation from the true electropherogram reaches a maximum around the length of the wrong marker peak. Although a previous normalization strategy for HiCEP (a cDNA-AFLP-based expression profiling technique) data analysis [12] can correct this kind of inappropriate fragment sizing, slight variations in the lengths of identical TDFs across different electropherograms still remain. Even if the variations of individual TDFs across electropherograms are very small (e.g., within 1 bp), cumulative errors of fragment sizing interfere with accurate alignment of identical TDFs and make visual evaluation troublesome. Therefore, the minimization of variations of identical TDFs is a prerequisite for accurate alignment and easy visual evaluation.

The purpose of the present study is the identification of differentially expressed TDFs from HiCEP time-course data using a method (called "GOGOT") proposed here. This is essentially the purpose of microarray analysis. However, the bottleneck is the construction of an expression matrix of TDFs (rows) per time points (columns) of HiCEP electrophoretic data due to the problem of imperfect alignment, though most microarray analysis uses such matrices as given data (e.g., [17,18]). GOGOT constructs an expression matrix consisting of valid TDFs whose alignment accuracies are objectively high and ranks TDFs according to their degrees of differential expression using a special statistic. The performance of GOGOT is demonstrated by analyzing a large set of HiCEP time-course data obtained from mouse embryonic stem (ES) cells.

## Results and discussion

A total of 256 primer combinations (16 MspI-NN primers combined with 16 NN-MseI primers; N = {A, C, G, T}) of HiCEP time-course data (mouse embryonic stem cells at 0, 12, 24, 48, and 96 h after adding stimulation for differentiation) was analyzed. HiCEP samples were technically duplicated and thus designated as *0h-1*, *0h-2*, *12h-1*, *12h-2*, *24h-1*, *24h-2*, *48h-1*, *48h-2*, *96h-1*, and *96h-2*. The data were preprocessed by a method which corrects fragment sizing errors caused by the mis-selection of size marker peaks [12].

**Figure 1:**
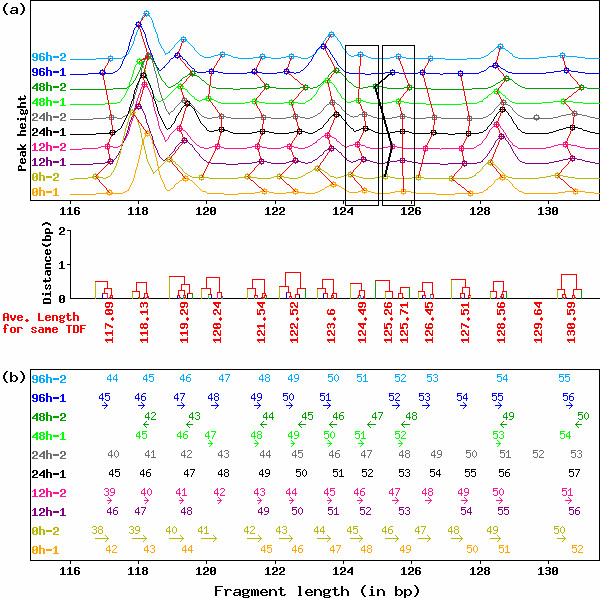
**Typical example of HiCEP electropherograms before normalization of peak fragment lengths by GOGOTnormL**. (a) Peak alignment of HiCEP electrophoretic data without GOGOTnormL normalization (upper) and the dendrograms obtained from complete-linkage clustering of the peak alignment (lower). Peaks connected by red lines and black bold lines are regarded as identical TDFs by the clustering-based peak alignment technique. Note that peak alignment subjectively failed in the range (124–126 bp) and that visual evaluation is also difficult because of the high variation in fragment lengths for individual TDFs. (b) Values of correction terms calculated by GOGOTnormL. For each serially numbered peak, directions and magnitudes are represented as arrows.

The remaining slight variations in the lengths of subjectively identical TDFs across different electropherograms would be sufficient to make visual evaluation tedious and tends to cause imperfect alignment of the same peaks across different electropherograms (such as shown in Fig. 1a). In this work, we applied the current method to each of the 256 sets (primer combinations).

To both improve the alignment and make visual evaluation less difficult, we adopted a method (called GOGOT) for the HiCEP expression analysis. Briefly, the procedure consists of four steps: (1) normalization of peak fragment lengths, (2) peak alignment, (3) normalization of peak heights, and (4) identification of differentially expressed TDFs. In our experience, step 1 is peculiarly important for easy visual evaluation, especially when the number of electropherograms being compared is increased, regardless of successful or unsuccessful peak alignment [12,19].

**Figure 2:**
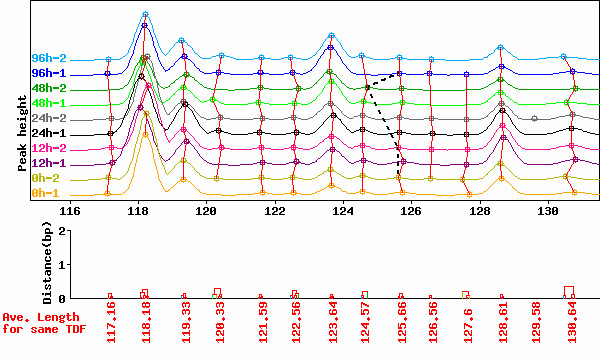
**Normalized peak fragment lengths in HiCEP electropherograms in Fig. 1**. Note that individual TDFs are represented by tight clusters and all peaks in the cluster are of course correctly aligned. The alignment connected by black bold lines in Fig. 1a is represented by black dashed lines and sectioned when peak alignment is reapplied to the normalized electropherograms.

In this paper, peak alignment for HiCEP electrophoretic data (Step 2) is performed using an algorithm based on complete linkage hierarchical clustering [20], though algorithms based on dynamic programming (DP) have widely been used for the purpose [21-25]. Perhaps a sophisticated DP-based method could perform accurate alignment such as shown in Fig. 2 for electropherograms in Fig. 1 without step 1. Nevertheless, the results of peak alignment for normalized electropherograms such as shown in Fig. 2 obtained from our two-step process (step 1 and 2) were satisfactory and those visual evaluations were very easy. The advantageous characteristics of our two-step approach over conventional DP-based methods [21-25] may be (i) easy visual evaluation by virtue of step 1 and (ii) easy traceability of why peaks are merged into a single TDF by virtue of a simple clustering-based method at step 2 (for details, see Methods). In general, labor-intensive visual evaluation of the electropherograms imposes bottlenecks on high-throughput expression analysis by electrophoretic methods including cDNA-AFLP [23]. Although there is currently no convincing rationale for choosing between the different methods, our two-step approach may eventually increase throughput. We demonstrate the feasibility of GOGOT in the rest of this section.

### Normalization of peak fragment lengths and its effect to peak alignment (Step 1 and 2)

Let $F_{i}^{k}$ be the *i*^th ^TDF (*i *= 1,..., *n*_*k*_) in electropherogram *P*^*k *^(*k *= 1,..., *m*; in this case *m *= 10). A TDF *F *is characterized by its length *L *(in bp), peak height *H*, and area *A *(in arbitrary units). The input data can be represented as 256 sets of (*L*_*i*_, *H*_*i*_, *A*_*i*_)^*k*^. Each electropherogram can be approximated by a Gaussian kernel using the input data [21-24]. The approximate electropherogram *P*(*t*), which is composed of *n *fragments *F*_*i *_(*i *= 1,..., *n*), at length *t *(in bp) is given by

$P(t)=\underset{i=1,2,...,n}{\max}\left[\frac{A_{i}}{\sigma_{i}{\left(2\pi \right)}^{1/2}}\exp\left\{-\frac{{\left(t-L_{i}\right)}^{2}}{2\sigma_{i}^{2}}\right\}\right]$, where *σ*_*i *_= *A*_*i*_/(*H*_*i*_$\sqrt{2\pi }$), the standard deviation of a Gaussian curve approximated to the shape of the *F*_*i*_.

As demonstrated in Fig. 1a, the direct application of clustering-based peak alignment (step 2) to HiCEP electrophoretic data does not work well because of the variation in the lengths of subjectively identical TDFs across electropherograms. In our opinion the four peaks aligned with black bold lines do not originate from identical TDFs and should be merged into the neighboring TDFs so that the peaks in each black box are aligned as identical TDFs. Furthermore, variant peaks across electropherograms which can make visual evaluation tedious still remain even if identical peak alignment could be performed by a sophisticated DP-based algorithm.

To this end, we first developed a method (called GOGOTnormL), which corrects peak fragment lengths *L *across electropherograms, so that the corrected lengths *L' *for subjectively identical TDFs are close to each other. The output data is represented as (*L'*, *H*, *A*)^*k*^, where *L' *is defined as *L' *= *L *- *c *and the correction term *c *is calculated using a moving window approach (see Methods).

Fig. 1b shows the directions ("←" for *c *> 0 and "→" for *c *< 0) and magnitudes (|*c*|, represented by the length of the arrow) for the correction of peak fragment lengths *L*. In this figure, arrows are assigned to fragments having |*c*| > 0.10, and GOGOTnormL correction is mainly performed for one electropherogram (*P*^48*h*-2^) to the short side and four electropherograms (*P*^0*h*-2^, *P*^12*h*-2^, *P*^48*h*-1^, and *P*^96*h*-1^) to the long side. Although the other lanes (*P*^0*h*-1^, *P*^12*h*-1^, *P*^24*h*-1^, *P*^24*h*-2^, and *P*^96*h*-2^) in the figure are of course slightly corrected (the respective average correction terms were 0.05, -0.04, 0.06, 0.06, and 0.01), they are used as references.

Fig. 2 shows the result of Fig. 1 after normalizing by GOGOTnormL. Visually, the electropherograms are normalized nearly perfectly. The average correlation coefficients among the ten electrophoretic patterns in the range shown in the figure before and after normalization are 0.79 and 0.91, respectively. The alignment consisting of the four questionable peaks discussed above (shown as black dashed lines in Fig. 2) disappears when clustering-based peak alignment (see Methods) is applied to the normalized electropherograms. Nevertheless, objective evaluation of the peak alignment for the electropherograms after GOGOTnormL normalization compared to that before normalization is difficult in practice and the goodness of peak alignment is judged by subjective visual evaluation. Although we believe the strategy of correcting for peak fragment lengths before peak alignment can increase the accuracy of peak alignment and make visual evaluation of aligned peak sets easier, some researchers might not agree.

GOGOTnormL can be regarded as an image warping method for adjusting different mobilities among corresponding peaks. There are some image warping methods for 1-D electrophoretic data produced by various experimental technologies such as single-stranded conformational polymorphism (SSCP) or pulsed-field gel electrophoresis (PFGE) [19,25-27]. However, the comparison between these methods and the GOGOTnormL is difficult in practice because of (i) different frameworks such as input data format, (ii) the subjectivity caused by visual evaluation of normalized electropherograms and aligned peaks, and (iii) a multitude of adjustable parameters.

The effectiveness of GOGOTnormL (Step 1) depends on the choice of the parameters *T *(the number of consecutive fragments as a "window" for the normalization; see equation 1 in the Methods section) and *D *(the empirically estimated maximum variation in the lengths of subjectively identical TDFs). The magnitude (|*c*|) of the correction term *c *for making the corrected length *L' *tends to decrease when *T *is large or *D *is small. In this case GOGOTnormL is ineffective since *L' *approaches *L*. On the other hand, when *T *is small or *D *is large GOGOTnormL is likely to produce erroneous results such as *L*_*i*_' > *L*_*i*+1_' (the size relationship must always be *L*_*i*_' <*L*_*i*+1_' regardless of normalization). Indeed, we observed such unfavourable cases when for example *T *= 3 and *D *= 20. Although we conservatively give *T *= 8 as the minimum number for which the size discrepancy disappears for all 256 sets of the HiCEP data (and *D *= 2 bp empirically), it is variable for each of the 256 sets used here and the other datasets. Similarly, the effectiveness of peak alignment via clustering (Step 2) depends on the choice of the parameter *u *which specifies the maximum difference of lengths for the aligned fragments. The parameter value *u *= 2 was chosen to specify the maximum variation among fragment lengths originating from TDFs determined (by eye) to be identical. Determination of parameter settings is ultimately the subjective decision of the researcher.

### Normalization of peak heights (Step 3)

Since cDNA-AFLP analysis handles small volumes of samples, problems during electrophoretic analysis such as over- or under-loading of samples cause variations in the overall peak heights *H *among different samples (or runs). Accordingly, normalization is essential when comparing cDNA-AFLP electrophoretic data.

**Figure 3:**
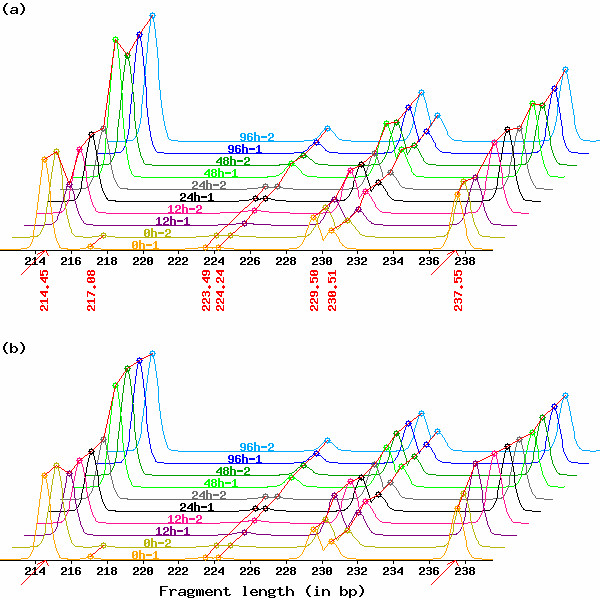
**Effect of peak height normalization by GOGOTnormH**. Electropherograms when peak height normalizations are performed using (a) all the reported TDFs (a conventional method used in [12, 28]) and (b) a subset of the selected TDFs (GOGOTnormH).

One simple approach is to assume that the average peak height of all the reported TDFs among samples is approximately the same [12,28]. It is formulated as $\sum_{i=1}^{n_{k}}H_{i}^{k}$ = ***constant ***for a set of electropherograms *P*^*k *^(*k *= 1,..., *m*). However, this approach sometimes fails because it includes two kinds of questionable peaks [22]. One is peaks near a preset detection limit, resulting in some peaks being detected and others not (for example, two peaks at 217 bp and four at 223.5 bp in Fig. 3). The other is peaks incorrectly identified as either broad peaks or two overlapping peaks because of the similar appearance of these two types.

We selected TDFs (or peaks) satisfying the following three conditions for peak height normalization: (1) peaks corresponding to an identical TDF exist in all samples, (2) they are not close to neighboring TDFs, and (3) the quality scores [12] assigned to each peak are high (for details, see the Methods section). We performed the normalization by adjusting the average peak heights of the selected TDFs among samples (we call the procedure GOGOTnormH for convenience) by assuming that only a minority of the selected TDFs display temporal expression changes. In general, the more selected TDFs you use for normalization, the more reliable the analysis. If you relax the standards for choosing TDFs, however, you compromise the reliability of the selected TDFs and the resulting peak alignment is less accurate. It's a tradeoff.

Peak height after normalization (*H'*) is defined as *H' *= *H *× *N*, where *N *is a normalization factor. GOGOTnormH outputs (*L'*, *H'*, *A*)^*k *^from the input dataset (*L'*, *H*, *A*)^*k *^(*k *= 1,..., *m*). Fig. 3 demonstrates the effect of GOGOTnormH (peak height normalizations are performed using all the reported TDFs in Fig. 3a and a subset of the selected TDFs in Fig. 3b). In Fig. 3, two TDFs indicated by red arrows satisfy the three conditions above. Since two electropherograms in each time point (e.g., *12h-1 *and *12h-2*) are the technical replicates, we can measure the power of GOGOTnormH (Fig. 3b) with the conventional method [12,28] (Fig. 3a) in light of the reproducibility in peak heights *H' *between the replicates. In comparison with electropherograms normalized using the conventional method [12,28] (Fig. 3a), we observed higher reproducibility in peak heights *H' *between replicate experiments in the normalized electropherograms (Fig. 3b): Peak heights *H' *in *12h-1 *(and *48h-2*) after conventional normalization are consistently lower than those in *12h-2 *(and *48h-1*) in electropherograms.

**Figure 4:**
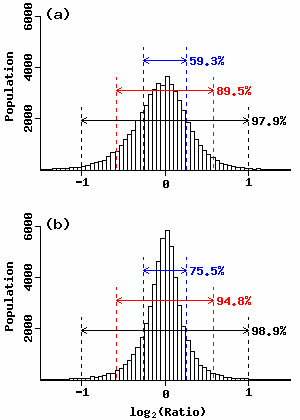
**Distribution of peak height ratios between replicate experiments**. Ratios are calculated using peak heights when the normalizations are performed using (a) all the reported TDFs (a conventional method used in [12, 28]) and (b) a subset of the selected TDFs (GOGOTnormH). Dashed lines in blue, red, and black indicate 1.2-, 1.5-, and 2.0-fold differences in peak heights, respectively.

We next show the statistics about ratios of peak heights between replicate experiments (Fig. 4). In the analysis of 256 sets (primer combinations) of HiCEP data, a total of 10,624 TDFs were used for peak height normalization and each TDF has five time points (0 h, 12 h, 24 h, 48 h, and 96 h). We observed a smaller dispersion for 53,120 (10,624 × 5) expression ratios after GOGOTnormH normalization. For example, there were 75.5% (or 94.8%) of 53,120 ratios satisfying ≤ 1.2 (or 1.5) fold difference after GOGOTnormH normalization, compared to 59.3% (or 89.5%) after normalization using the conventional method [12,28]. These results demonstrate the validity of our strategy at least for peak height normalization of the 10,624 TDFs. The minimization of differences between technical replicates is, of course, one quality criterion and it remains unclear how efficiently both algorithms reveal genuine temporal expression changes. The comparison of our GOGOTnormH and other conventional methods on real data which contain genuinely induced/suppressed TDFs is one of the next important task.

Although we here calculated the normalization factor *N *using the average peak height of the selected TDFs (GOGOTnormH) to compare the effect of different sets of TDFs, there are many other possible approaches for calculating the normalization factor *N *such as the median, the trimmed mean [29], Tukey's one-step biweight method [30], and so on. Further improvement in the choice of those methods as well as the selection of valid TDFs remains to be studied.

### Identification of differentially expressed TDFs (Step 4)

We have an expression matrix (called the "HiCEP expression matrix") consisting of 10,624 TDFs and ten temporal samples obtained from HiCEP electrophoretic data. We searched through these 10,624 TDFs looking for differentially expressed TDFs, though there are many others in the original data (such as the four TDFs in the range 222–232 bp shown in Fig. 3). This is because the 10,624 TDFs have two advantages: (1) they have high reproducibility between replicate experiments (Fig. 4) and (2) their annotation is potentially easy by virtue of the above three conditions used in GOGOTnormH normalization.

**Figure 5:**
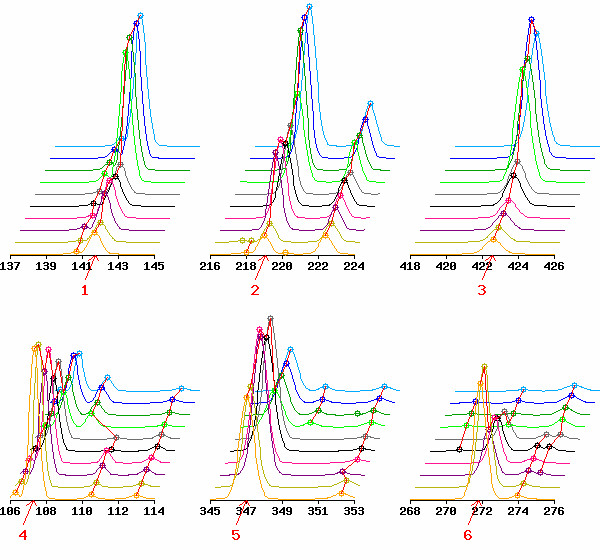
**Expression patterns of top six TDFs listed in Table 2**. Local (8 bp of range) electropherograms including the top-ranked TDFs indicated by red arrows are shown. Numbers below arrows indicate the ranks of the TDFs. Each electropherogram is shown in common scaling.

We used a special statistic called GOGOTstat (equation 4) for the detection of differentially expressed TDFs (see Methods). The statistic gives a nonnegative score, with the value 0 for TDFs expressed uniformly in all the interrogated samples and a high score for differential expression. Of course, there are many ways to rank TDFs according to the degrees of their differential expression. For example, a *t*-like statistic (equation 5) obtained by substituting the standard deviation of peak heights in replicate experiments for the ratio in equation 4 can be considered. However, such a *t*-like statistic often gives a high score (rank) to questionable TDFs whose overall peak heights are quite low (Additional file 1). This high score is mainly caused by a low value for the denominator in the *t*-like statistic. We do not give high scores to these questionable TDFs for the following two reasons. One is they have high relative error (low signal-to-noise ratio). In general, relative error increases for low peak heights when the peak height approaches the background level [31]. The reliability of such candidates is thus implausible [32]. The other reason is the disagreement with visual evaluation (Additional file 2 and Fig. 5). Unlike in microarray analysis, the true significance of the candidate patterns obtained from high-dimensional electrophoretic data such as HiCEP or Differential Display must be confirmed visually. It is important to develop a score metric compatible with visual evaluation. Our statistic (GOGOTstat) is a straightforward application of this idea.

Table 1 lists expression data (peak heights) and the statistics for the top ten TDFs. As expected, there is a wide range of peak heights across time points and high reproducibility between the replicates for each time point. Visual evaluation of those local electrophoretic patterns also demonstrates that peak alignment is correctly performed (see Fig. 5). The expression data (peak heights) and two statistics (GOGOTstat and *t*-like statistic) for all 10,624 TDFs in the HiCEP expression matrix are available in Additional file 1.

**Table 1:** Expression data for top ten TDFs ranked by GOGOTstat. Statistic: score of GOGOTstat. The values of peak heights after GOGOTnormH normalization are shown.

Rank	Peak height										Statistic
		
	*H*^0*h*-1^	*H*^0*h*-2^	*H*^12*h*-1^	*H*^12*h*-2^	*H*^24*h*-1^	*H*^24*h*-2^	*H*^48*h*-1^	*H*^48*h*-2^	*H*^96*h*-1^	*H*^96*h*-2^	
1	134	121	228	236	183	186	811	828	843	817	134.7
2	115	115	483	489	388	425	554	870	881	873	134.0
3	91	88	105	112	189	201	704	698	868	706	129.0
4	938	894	650	710	455	485	214	237	295	233	123.7
5	636	627	865	835	712	756	231	247	248	261	116.8
6	719	752	285	282	203	172	16	12	16	10	116.6
7	803	811	320	332	293	299	188	203	212	180	114.9
8	141	153	335	353	342	338	743	763	646	774	112.7
9	643	627	600	634	560	527	90	84	65	72	106.5
10	684	704	650	665	572	666	149	133	132	136	104.4

Since the detection of differentially expressed TDFs is based on the HiCEP expression matrix, we can estimate the false discovery rate (FDR), defined as the expected proportion of false positives among true differentially expressed TDFs [33]. The random permutation test suggests that tens of top-ranked TDFs have statistical significance at low (5–10%) FDR (see Table 2). Visual evaluation confirmed the validity of the differential expression patterns for the top 100 TDFs and the non-differential patterns for the last 100 TDFs (data not shown except for the top six TDFs).

**Table 2:** Numbers of TDFs called significant at various thresholds.

Statistic	Randomized	Observed	FDR
13.4	1017.8	2037	50%
20.2	481.9	1202	40%
30.0	163.2	543	30%
40.1	53.9	270	20%
57.2	8.3	83	10%
76.5	1.4	28	5%

Statistic: score of GOGOTstat satisfying given FDRs.

Randomized: average number called significant by analyzing 1,000 randomly permutated HiCEP expression matrices.

Observed: number called significant by analysing the original HiCEP expression matrix of 10,624 TDFs and 10 samples.

The FDR is defined as the percentage of falsely significant TDFs compared to the TDFs called significant.

Note that there must be other differentially expressed TDFs (i.e., true-negative TDFs) which are not included in the dataset (i.e., the 10,624 TDFs in the expression matrix) because they do not satisfy the above three conditions used in Step 3. For example, we cannot identify differentially expressed TDFs if peaks constituting an identical TDF are not detected due to the effect of a preset detection limit, with the current settings used for the selection of the 10,624 TDFs. Of course, they should be detected if they are genuine. However, as also discussed in the selection of the 10,624 TDFs, the more true-negatives you want to detect, the more tedious visual inspections you have to do. It's a tradeoff.

In practice one may want to detect the differentially expressed TDFs having no data at time point *k *due to reasons such as the above, though the current analysis does not analyze those TDFs. One way to deal with them would be to let *H*^*k *^= *constant *(e.g., the predetermined value of the peak detection threshold). Of course, there are many possible ways to analyze those TDFs. Further improvement of GOGOTstat to make it universal remains to be done.

## Conclusion

We propose an integrated strategy (called GOGOT) for identifying differentially expressed TDFs from HiCEP time-course data. As with mass spectrometry data, there remains the problem that the same peaks across electropherograms are not perfectly aligned in general [34,35]. We demonstrated that the application of GOGOTnormL (step 1) dramatically contributes to successful peak alignment via clustering (step 2) and facilitates visual evaluation (Figs. 1 and 2). This enabled us to construct a HiCEP expression matrix consisting of 10,624 valid TDFs and ten samples from a total of 256 sets of ten HiCEP electrophoretic data samples. Normalization of peak heights in the matrix using GOGOTnormH increased reproducibility between replicate experiments (Figs. 3 and 4). These results facilitate the use of various analysis methods for the identification of differentially expressed genes in microarray data.

We used a simple statistic (GOGOTstat at step 4) to rank TDFs according to the degree of their temporal expression change. Researchers involved in HiCEP analysis were very satisfied with the ranking of the differentially expressed TDFs (Fig. 5). Although the current statistic was developed for analyzing HiCEP data, it can also be applied to microarray data since the input data (expression matrix) is the same. As future work, it would be interesting to evaluate the potential of the statistic in the analysis of microarray data.

The current method GOGOT can be regarded as a method for analyzing 1-D electrophoretic data. There are a number of methods for analyzing 1-D electrophoretic data produced by various experimental technologies [19,21-29]. Compared to them, GOGOT can be positioned a method specialized for cDNA-AFLP data analysis. The fully automated GOGOT procedure dramatically increased the throughput of data analysis (approximately, 20–30 times). We also verified the power of the strategy using other HiCEP datasets (data not shown). In addition to cDNA-AFLP data, the algorithm should be easily applicable to other one-dimensional electrophoretic data such as Differential Display or AFLP. GOGOT can be a powerful tool for detecting differentially expressed TDFs from multiple one-dimensional electrophograms.

## Methods

### Data

Samples were prepared from mouse embryonic stem (ES) cells at 0, 12, 24, 48, and 96 h after removal of leukemia inhibitory factor (LIF) from the culture medium. The samples subjected to HiCEP reaction were technically duplicated (i.e., the replicates were from the same samples). We thus designated each sample as *0h-1*, *0h-2*, *12h-1*, *12h-2*, *24h-1*, *24h-2*, *48h-1*, *48h-2*, *96h-1*, and *96h-2*. The HiCEP reaction was performed according to a previous report [8]. Most of the steps are the same as in standard AFLP [2] except for (i) the primers, whose GC content is 55% to 60% and (ii) the annealing temperature (71.5°C) in selective PCR [8].

cDNA prepared from mRNA extracted from each sample were digested with two 4-bp-cutting endonucleases (*Msp*I combined with *Mse*I) and ligated with the corresponding adaptors. The resulting HiCEP templates, 5'-*Msp*I-*Mse*I-3' fragments, were amplified using fluorescently labeled primers. In total, 256 primer combinations (16 *Msp*I-NN primers combined with 16 NN-*Mse*I primers; N = {A, T, G, C}) were used in the HiCEP analysis. The details of the protocol of the HiCEP reaction are described elsewhere [8].

The PCR products were denatured and loaded on an ABI Prism 310 (Applied Biosystems) for capillary gel electrophoresis. The digitized images were noise-reduced and baseline-corrected by the GeneScan software (Applied Biotech). After noise reduction and baseline correction, the software quantifies each TDF *F *by fragment length *L *(in bp), peak height *H*, and area *A *(in arbitrary units) in the size calibration range (35–700 bp in this analysis). Accordingly, the data obtained from a HiCEP electropherogram consists of a collection of TDFs and each TDF (or peak) $F_{i}^{k}$ (*i *= 1,..., *n*_*k*_) in electropherogram *P*^*k *^(*k *= 1,..., *m*; *m *= 10 in this case) is characterized by (*L*_*i*_, *H*_*i*_, *A*_*i*_)^*k*^, where the peaks are originally numbered with respect to their sizes.

To correct fragment sizing errors caused by the mis-selection of size marker peaks, the preprocessing method of Kadota et al. [12] was adopted. Accordingly, the variation in the lengths of subjectively identical TDFs in the input data was small (± 1 bp at most; see Fig. 1).

### Normalization of peak fragment lengths (Step 1; GOGOTnormL)

The purpose of step 1 using GOGOTnormL is to correct peak fragment lengths *L *across electropherograms so that the corrected lengths *L' *for subjectively identical TDFs are close to each other. The output data for the *m *electropherograms (*k *= 1,..., *m*) to be compared is represented as (*L'*, *H*, *A*)^*k*^. The normalization is performed for each electropherogram *P*^*k *^(*k *= {1,..., *m*}) using a moving window approach. Briefly, the procedure is as follows:

### Step 1-1. Determination of the window (range) in the target electropherogram

Each electropherogram can be approximated by a Gaussian kernel using the input data (*L*, *H*, *A*). The approximate electropherogram *P*(*t*), which is composed of *n *fragments *F*_*i *_(*i *= 1,..., *n*), at length *t *(in bp) is given by

$P(t)=\underset{i=1,2,...,n}{\max}\left[\frac{A_{i}}{\sigma_{i}{\left(2\pi \right)}^{1/2}}\exp\left\{-\frac{{\left(t-L_{i}\right)}^{2}}{2\sigma_{i}^{2}}\right\}\right]$, where *σ*_*i *_= *A*_*i*_/(*H*_*i*_$\sqrt{2\pi }$), the standard deviation of the Gaussian curve approximated to the shape of the *F*_*i*_.

A total of (*n*_*target *_- *T *+ 1) ranges are defined from a target electropherogram *P*^*target *^(*target *= {1,..., *m*}), where *n*_*target *_is the number of fragments in *P*^*target*^. The *i*^th ^(*i *= 1,..., *n*_*target *_- *T *+ 1) range consists of *T *fragments *F*_*a *_(*a *= *i*,..., *i*+*T*-1). In this analysis, we let *T *= 8 though other numbers are of course possible. For example, the first range consists of eight fragments *F*_1_, *F*_2_,..., *F*_8 _and the (*n*_*target *_- *T *+ 1)^th ^range consists of $F_{n_{target}-7}\text{, }F_{n_{target}-6}\text{,}...\text{, }F_{n_{target}}$. The *i*^th ^range in the target electropherogram, (*start*_*i*_-*end*_*i *_bp)^*target*^, is given by:

(1) $$\begin{array}{l} start_{i}=\{\begin{array}{ll} L_{i}-(L_{i}-L_{i-1})/2 & if\;L_{i}-2\sigma_{i}<L_{i-1}\;and\;i>1,\\ L_{i}-2\sigma_{i} & else, \end{array}\\ end_{i}=\{\begin{array}{ll} L_{i+T-1}+(L_{i+T}-L_{i+T-1})/2 & if\;L_{i+T-1}+2\sigma_{i+T-1}>L_{i+T}\;and\;i<n_{target}-T,\\ L_{i+T-1}+2\sigma_{i+T-1} & else, \end{array} \end{array}$$

### Step 1–2. Selection of the reference for each range

The selection of the reference electropherogram (a kind of typical electrophoretic pattern) for the normalization of the *i*^th ^range (*start*_*i*_-*end*_*i *_bp)^*target *^is performed according to Kadota et al. [12]. Briefly, quality scores *Q*($L_{i}^{k}$) at fragment lengths $L_{i}^{k}$ in electropherogram *P*^*k *^(*k *= {1,..., *m*}) are estimated by a windowing calculation of local average correlation coefficients between *P*^*k *^and the other electropherograms (for details, see [12]). A high (or low) score for electropherogram *P*^*k *^indicates a high (or low) level of relative similarity between the electropherogram and the others at around length $L_{i}^{k}$. The reference to the *i*^th ^range (*start*_*i*_-*end*_*i *_bp)^*target *^is the electropherogram *P*^*k *^(*k *= {1,..., *m*}) satisfying both (i) the number of peaks satisfying ***start***_*i *_≤ $L_{a}^{k}$ ≤ ***end***_***i ***_is *T*/2 or more and (ii) the average quality score $\bar{Q\left(L_{a}^{k}\right)}$ is the maximum.

### Step 1–3. Normalization of the target electropherogram

For the *i*^th ^(*i *= 1,..., *n*_*target *_- *T *+ 1) range (*start*_*i*_-*end*_*i *_bp), GOGOTnormL searches for the sub-electropherogram *P*^*target *^[*start*_*i*_+*x*_*i*_, *end*_*i*_+*x*_*i*_] (in the target electropherogram) that is most similar to the reference *P*^*reference *^[*start*_*i*_, *end*_*i*_] around the range. The most similar sub-electropherogram is the one that achieves the highest correlation coefficient *r*_*i *_between *P*^*reference *^[*start*_*i*_, *end*_*i*_] and the various candidates *P*^*target *^[*start*_*i*_+*x*, *end*_*i*_+*x*] (-*D *<= *x *<= +*D*). *x*_*i *_is the *x *at the highest correlation coefficient *r*_*i*_. We set *D *= 2, as a maximal realistic displacement.

If we use a moving window of *T *fragments, most fragments (*n*_*target*_-2*T*+2 fragments) $F_{i}^{target}$ (*i *= *T*, *T*+1,..., *n*_*target *_- *T*+1) have *T *values of *x*_*a *_and *r*_*a *_(*a *= *i*-*T*+1, *i*-*T*+2,..., *i*).

*L*_*i*_' (*L*_*i *_in the target electropherogram after normalization) is obtained from *L*_*i*_' = *L*_*i *_- *c*_*i*_. The correction term *c*_*i *_is calculated by using *x*_*a *_and *r*_*a *_(*a *= *i*-*T*+1, *i*-*T*+2,..., *i*):

(2) $$c_{i}=\frac{\sum_{a=i-T+1}^{i}x_{a}\times w(r_{a})}{\sum_{a=i-T+1}^{i}w(r_{a})},$$

where *w*(*r*_*a*_) is the tricube weight function of *r*_*a*_, namely *w*(*r*_*a*_) = 1 - (1 - *r*_*a*_^3^)^3^.

### Peak alignment via clustering (Step 2)

The major difficulty in one-dimensional (1-D) electrophoretic data (including HiCEP) analysis is the alignment of multiple peak sets. In order to solve this problem, we here use complete-linkage hierarchical clustering, since the strategy was successfully applied to mass spectrometry data by Tibshirani et al. [20]. To our knowledge, it is the first time clustering-based peak alignment has been used for 1-D electrophoretic data analysis.

The absolute difference in fragment lengths between two peaks is used as the distance. To guarantee that each cluster represents individual TDFs, two clusters are merged only when all the peaks in the two clusters are derived from different samples. Since we analyze ten samples, the maximum peak number in each cluster is thus ten. The dendrogram is cut off at height *u *bp. In this study, we set *u *= 2, implying that every peak in the cluster is at most 2 bp from any other peak in that same cluster by virtue of the use of complete-linkage.

As previously mentioned by Tibshirani et al. [20], the idea for peak alignment is that tight clusters should represent identical TDFs. The use of clustering-based peak alignment combined with the correction of peak fragment lengths by GOGOTnormL was successful (Figs. 1 and 2).

### Normalization of peak heights (Step 3; GOGOTnormH)

As shown in Fig. 3a, the conventional normalization strategy [12,28], in which average peak heights of all the reported TDFs among samples are adjusted, sometimes does not work well. We assert the reason is the use of all the reported TDFs in individual samples. GOGOTnormH selects TDFs satisfying the following three conditions:

(i) peaks corresponding to identical TDFs are exhibited by all samples. The idea is essentially the same as that of Fushiki et al. [35]: peaks exhibited by only a few samples may just be noise, but peaks exhibited by many subjects appear to be true TDFs.

(ii) the neighboring TDFs are not so close. Suppose that (a) individual TDFs in a set of electropherograms are numbered with respect to their average sizes, (b) there are *m *(10 in this case) peaks in the *i*^th ^TDF (*i *= 1,..., *n*_*TDF*_) by condition (i), and (c) the ten peaks corresponding to the *i*^th ^TDF (*i *= 1,..., *n*_*TDF*_) in electropherograms *P*^*k *^(*k *= 1,..., *m*) are characterized by length $L_{TDF_{i}}^{k}$, peak height $H_{TDF_{i}}^{k}$, area $A_{TDF_{i}}^{k}$, and standard deviation $\sigma_{TDF_{i}}^{k}(=A_{TDF_{i}}^{k}/\left(H_{TDF_{i}}^{k}\sqrt{2\pi }\right))$. The width of the *i*^th ^TDF is defined as

$$[\min\{L_{TDF_{i}}^{1}-1.5\sigma_{TDF_{i}}^{1},...,\;L_{TDF_{i}}^{m}-1.5\sigma_{TDF_{i}}^{m}\},\max\{L_{TDF_{i}}^{1}+1.5\sigma_{TDF_{i}}^{1},...,\;L_{TDF_{i}}^{m}+1.5\sigma_{TDF_{i}}^{m}\}].$$

Finally, the TDFs satisfying following conditions are selected:

• Case *i *= 1,

$$\begin{array}{l} \max\{L_{TDF_{i}}^{1}+1.5\sigma_{TDF_{i}}^{1},...,\;L_{TDF_{i}}^{m}+1.5\sigma_{TDF_{i}}^{m}\}<\\ \min\{L_{TDF_{i+1}}^{1}-1.5\sigma_{TDF_{i+1}}^{1},...,\;L_{TDF_{i+1}}^{m}-1.5\sigma_{TDF_{i+1}}^{m}\} \end{array}$$

• Case *i *= 2,..., *n*_*TDF *_-1,

$$\begin{array}{l} \max\{L_{TDF_{i}}^{1}+1.5\sigma_{TDF_{i}}^{1},...,\;L_{TDF_{i}}^{m}+1.5\sigma_{TDF_{i}}^{m}\}<\\ \min\{L_{TDF_{i+1}}^{1}-1.5\sigma_{TDF_{i+1}}^{1},...,\;L_{TDF_{i+1}}^{m}-1.5\sigma_{TDF_{i+1}}^{m}\} \end{array}$$

and

$$\begin{array}{l} \min\{L_{TDF_{i}}^{1}-1.5\sigma_{TDF_{i}}^{1},...,\;L_{TDF_{i}}^{m}-1.5\sigma_{TDF_{i}}^{m}\}>\\ \max\{L_{TDF_{i-1}}^{1}+1.5\sigma_{TDF_{i-1}}^{1},...,\;L_{TDF_{i-1}}^{m}+1.5\sigma_{TDF_{i-1}}^{m}\} \end{array}$$

• Case *i *= *n*_*TDF*_,

$$\begin{array}{l} \min\{L_{TDF_{i}}^{1}-1.5\sigma_{TDF_{i}}^{1},...,\;L_{TDF_{i}}^{m}-1.5\sigma_{TDF_{i}}^{m}\}>\\ \max\{L_{TDF_{i-1}}^{1}+1.5\sigma_{TDF_{i-1}}^{1},...,\;L_{TDF_{i-1}}^{m}+1.5\sigma_{TDF_{i-1}}^{m}\} \end{array}$$

(iii) all of the quality scores *Q*($L_{TDF_{i}}^{k}$) assigned for the ten peaks corresponding to the *i*^th ^TDF are 0.7 or more. The score provides an objective goodness for the estimation of the fragment lengths $L_{TDF_{i}}^{k}$ and our previous study suggests the value of 0.7 is the minimum necessary for the accurate alignment of valid TDFs across electropherograms [12].

The peak height after normalization (*H'*) is obtained from *H' *= *H *× *N*, where *N *is a normalization factor. The normalization factor *N*^*k *^for electropherogram *P*^*k *^is calculated by

(3) Nk=100Hselectedk¯,

where $\bar{H_{selected}^{k}}$ is the average peak height for the selected TDFs in electropherogram *P*^*k*^. After normalization, the average peak height in each electropherogram is adjusted to 100.

### Identification of differentially expressed TDFs (Step 4; GOGOTstat)

The differentially expressed TDFs are detected from a total of 10,624 valid TDFs used for peak height normalization at step 3. Consider one expression vector ***H ***consisting of peak heights *H*^*p*-*q *^at the *q*^th ^replicate experiment (*q *= 1, 2,..., *n*_*p*_) in time point *p*. To quantify the degree of differential expression, we define a statistic GOGOTstat as

(4) $$GOGOTstat=\frac{\max(\bar{H^{p}})-\min(\bar{H^{p}})}{\sum_{p,q}\max(H^{p-q})/\min(H^{p-q})},$$

where $\bar{H^{p}}$ is the average of *n*_*p *_normalized peak heights at time point *p *and *H*^*p*-*q *^is the normalized peak height in the *q*^th ^replicate experiment at time point *p*. Since each TDF has ten normalized peak heights in five time points (i.e., *p *= (*0h*, *12h*, *24h*, *48h*, *96h*)) and each time point has two replicates (i.e., *n*_*p *_= 2), the generalized expression vector can be written as ***H ***= (*H*^0*h*-1^, *H*^0*h*-2^,..., *H*^96*h*-2^). For example, the statistic of the top-ranked TDF shown in Table 1 is calculated as follows:

$$\begin{array}{c} GOGOTstat=\frac{\max(127.5,\;232,\;184.5,\;819.5,\;830)-\min(127.5,\;232,\;184.5,\;819.5,\;830)}{\left(134/121)+(236/228)+(186/183)+(828/811)+(843/817\right)}\\ =\frac{830-127.5}{5.21}=134.7 \end{array}$$

The *t*-like statistic compared to our GOGOTstat statistic (see Results and discussion) is defined as

(5) $$t-likestatistic=\frac{\max(\bar{H^{p}})-\min(\bar{H^{p}})}{\sum_{p=0h,12h,24h,48h,96h}\sigma^{p}},$$

where *σ*^*p *^is the standard deviation of *n*_*p *_normalized peak heights at time point *p*.

## Competing interests

The author(s) declare that they have no competing interests.

## Authors' contributions

KK invented the method and wrote the paper. RA, YN, and MA provided critical comments and led the project.

## Supplementary Material

Additional File 1 **Expression data for 10,624 valid TDFs**. Peak heights after GOGOTnormH for 10,624 valid TDFs are provided. It also contains two statistics measured by GOGOTstat and a *t*-like statistic and their ranks. Click here for file

Additional File 2 **Expression patterns of top six TDFs ranked by a *t*-like statistic**. Legends are the same as given in Fig. 5. Click here for file
