# Supplementary figures and images for: GOGOT: a method for the identification of differentially expressed fragments from cDNA-AFLP data
Source: Algorithms Mol Biol. 2007 May 30;2:5. doi: 10.1186/1748-7188-2-5 (PMC1904450; doi:10.1186/1748-7188-2-5)

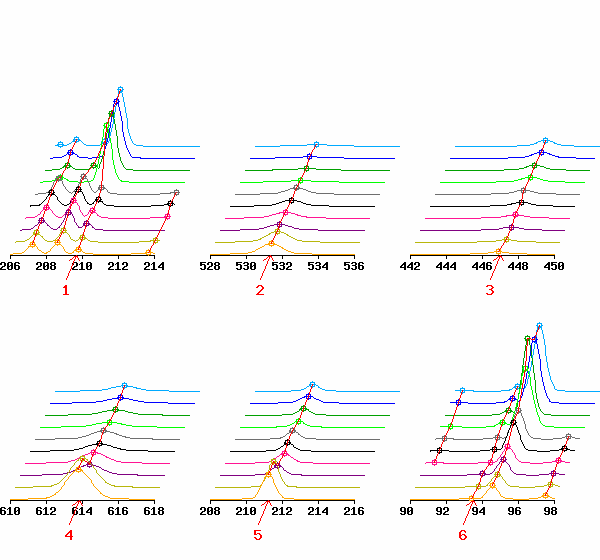

Supplement: Additional File 2 — Expression patterns of top six TDFs ranked by a t-like statistic. Legends are the same as given in Fig. 5. [file 1748-7188-2-5-S2.png]
